# Anatomic Reversal of Shoulder Joint After Neck of Humerus Fracture

**DOI:** 10.7759/cureus.59672

**Published:** 2024-05-05

**Authors:** Alysid Fernandes, Eugine Chizooma, Oliver Pearce, Richard Craig

**Affiliations:** 1 Trauma and Orthopaedics, Milton Keynes University Hospital, Milton Keynes, GBR

**Keywords:** post-menopausal osteoarthritis, orthogeriatrics, humeral shaft fracture, avascular necrosis humeral head, trauma and orthopedic surgery

## Abstract

We report on a singular case of a unique form of post-traumatic reversal of the humeral head after humeral neck fracture, in which the pattern of collapse resulted in the formation of a native reverse polarity shoulder. In essence, the humeral head became a socket, and the glenoid rounded to become a head with well-preserved shoulder function. To our knowledge, this is the first case of an acquired shoulder deformity that bears a remarkable functional similarity to a prosthetic reverse polarity shoulder replacement.

## Introduction

Reverse shoulder arthroplasty has been one of the most significant technical innovations in shoulder surgery in the past three decades. It is a surgical procedure used to treat certain shoulder conditions, particularly when there is a combination of severe rotator cuff deficiency, advanced arthritis or trauma [1]. Unlike traditional shoulder replacement surgery, which involves replacing the damaged ball-and-socket joint with a prosthetic ball on the humerus and a prosthetic socket in the scapula, reverse shoulder arthroplasty reverses the orientation of these components. Initially recommended for patients with rotator cuff arthropathy, surgeons have expanded its application to massive cuff tears without arthritis, fracture care, rheumatoid arthritis, and failed prior surgery replacements with a high level of success [2]. While the surgical procedure is widely accepted, little is known about the natural occurrence of a reverse shoulder joint. There are sparse reports of congenital reverse shoulder joint deformity in the literature [3,4].

We report a case of a reverse shoulder joint following a proximal humeral fracture. To our knowledge, this is the first case of an acquired reverse shoulder joint, where the humeral head became the “socket” and the glenoid became the “ball”.

## Case presentation

In 2004, a woman in her 70s presented to her local accident and emergency department with a right, displaced, valgus-impacted proximal humeral fracture following a fall. Despite considerable impaction of the humeral head, a non-operative treatment strategy was agreed upon. No records are available regarding the treatment, since it took place at another facility without digitised notes. 

**Figure 1:**
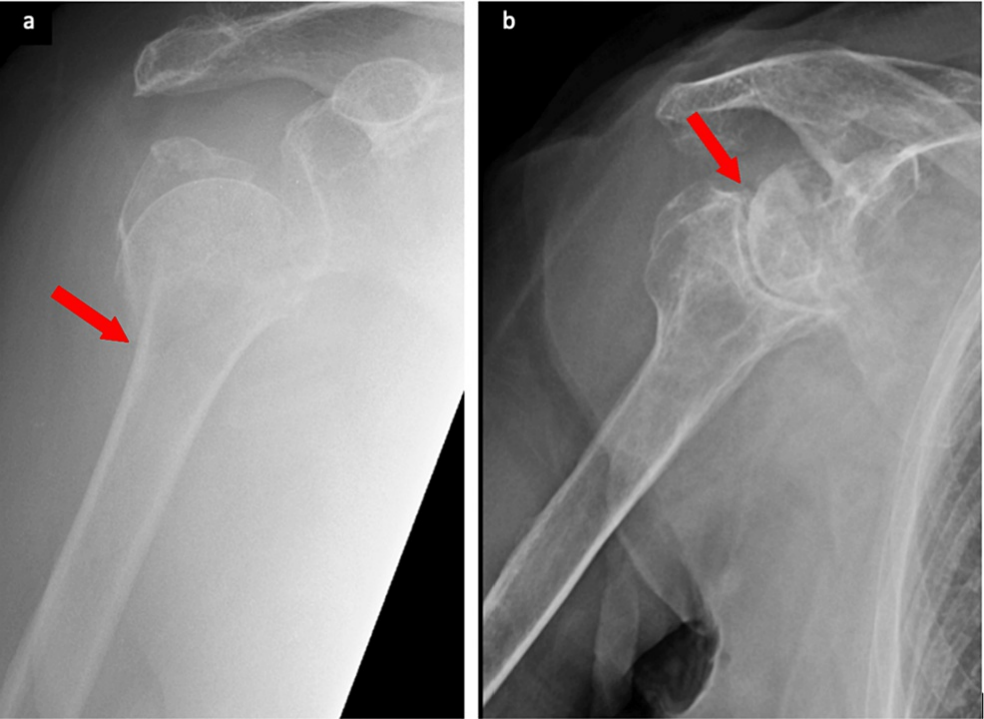
Patient’s (a) R shoulder in 2004, and (b) native, well-preserved, reversed R shoulder in 2022.

Years later, now in her 90s, the lady presented to our institution with a right neck femur fracture following a fall. Her past medical history included Alzheimer’s disease, hypertension, ischaemic heart disease, stage 3 chronic kidney disease, and basal cell carcinoma. An abnormal appearance of a native right reverse shoulder joint was found incidentally when a chest x-ray was done as part of the neck of femur admission protocol (Figure 1). The patient appeared to have unwittingly adapted to her novel shoulder anatomy as she did not exhibit any functional deficits or report any pain-related symptoms. Plans to investigate her reverse shoulder joint further were abandoned as the patient, unfortunately, passed away a few days later due to an unrelated illness.

## Discussion

While reverse total shoulder joint arthroplasty is a common procedure in orthopaedic practice, a native reverse shoulder joint remains a rare phenomenon. To our knowledge, only two cases of a naturally occurring reverse shoulder joint have been reported in the literature [3,4]. It is important to highlight that both patients had congenital deformities, which included isolated congenital proximal humeral hypoplasia, and thalidomide-induced dysmelia, respectively. The unusual glenohumeral joint anatomy in both cases is thought to have been the result of physiological adaptation processes that occurred to facilitate optimum function during development. 

Our case differs from these as the first report of a post-traumatic reverse shoulder. It suggests that the traumatic reversal of native shoulder anatomy may provide a similar level of adequate shoulder function as that enjoyed following reverse shoulder replacement surgery. The precise mechanisms that led to the development of a reverse shoulder joint in our patient are unclear. We surmise that the head fragment may have become further displaced, flipped, interposed and adherent to the native glenoid. However, the appearance may simply be the result of avascular necrosis and mechanical erosion over many years, resulting in pseudoarthrosis. As in reverse shoulder arthroplasty, the centre of rotation of the joint had moved inferior and medial, consistent with Grammont’s original design theory [5]. This affords the deltoid muscle an improved mechanical advantage to elevate and abduct the arm. We noted that the greater tuberosity had united in a fairly anatomical position to the shaft, which may have contributed to some preservation of posterior rotator cuff function.

We recognise that the records of her initial presentation with the original fracture are sparse. And that a more complete notes and radiology record would have strengthened this report. The patient herself on admission with her hip fracture, by this time had significant cognitive impairment, as well as a distracting injury. Nonetheless appearing none the worse in terms of both pain, as well as function in her novel post-traumatic shoulder anatomy.

## Conclusions

Naturally acquired reverse shoulders are a rare phenomenon. The presence of a naturally occurring reverse shoulder joint, and its similarity to the prosthetic reverse shoulder joint is an example of a type of convergent evolution. In this case, natural evolution converging with design innovation results in a similar structural and functional outcome. By reporting and analysing these unique instances, we gain valuable insights into the role reverse shoulder arthroplasty plays in addressing this specific type of injury.
